# Adverse drug reactions reported to the drug and poison information center of Tehran, Iran

**DOI:** 10.1371/journal.pone.0185450

**Published:** 2017-09-26

**Authors:** Fatemeh Saheb Sharif-Askari, Narjes Saheb Sharif-Askari, Mohammadreza Javadi, Kheirollah Gholami

**Affiliations:** 1 Clinical Pharmacy Department, Faculty of Pharmacy, Tehran University of Medical Sciences, Tehran, Iran; 2 Research Center for Rational Use of Drugs, Tehran University of Medical Sciences, Karimkhan Zand Avenue, Hafte Tir Square, Tehran, Iran; NETHERLANDS

## Abstract

**Background:**

Burden of adverse drug reactions (ADRs), in home-environment and domestic settings, is unknown.

**Objective:**

To discuss the epidemiology of reported ADRs to 13-Aban drug and poison information center (DPIC) and to discuss the burden of hospitalization caused by these ADRs from commonly implicated therapeutic groups.

**Methods:**

A retrospective analysis of the yellow card schemes of suspected ADRs reported to the 13-Aban DPIC was conducted from 21 March 2013 to 21 November 2016 inclusive.

**Main outcome measures:**

Characteristics of the ADRs, such as the sex and age of the patient, the therapeutic group involved, and the medical outcome of the exposure, were examined. ADR Hospitalization (ADRH) index was calculated for each drug group by dividing the number of ADR-related hospitalizations with total number of reported ADR cases (n = 748), and then multiplying by 100.

**Results:**

ADRs were reported for 748 patients representing 5 cases per 1000 enquiries to the 13-Aban DPIC over almost 4-years of the study period. Public were responsible for reporting every 4 out of 5 ADR cases (n = 651, 87%) and the remaining 1 out of 5 ADR cases was reported by the health care professionals (n = 97, 13%). Most of the ADRs had a medical outcome documented as having a minor effect or were minimally bothersome to the patients (n = 509, 68%), and less than 4.9% (n = 37) were documented as having a major effect or were life-threatening. Overall, 7.4% (n = 55) of ADRs were resulted in hospitalization. Antibacterials for systemic use represented the therapeutic group with the highest hospitalization index (1.7%).

**Conclusions:**

The study concluded that ADRs to antibiotics are common and some of them resulted in hospitalization.

## Introduction

Adverse drug reactions (ADRs) represent a major burden on healthcare systems and are a common cause of hospital admissions as well as of in-hospital morbidity and mortality [1–3]. Prompt reporting of ADRs is crucial for any pharmacovigilance system. In Iran this is managed through the yellow card scheme which is operated by the Iranian Adverse Drug Reaction Monitoring Center (IADRMC) at the Ministry of Health [4]. Information in these cards is the main source of data for the IADRMC and is mostly supplied by health professionals such as physicians, nurses, and pharmacists [4]. However, direct reporting of suspected ADRs by public, which is already well established in other countries [5–7], has shown to have many potential benefits. Public can report by telephone either to the IADRMC or to drug information centers. One of the main benefits for advocating direct public reporting is that suspected ADRs reported to physicians might not be passed on to the regularity authority, or even recorded in medical records. Moreover, the public tend to provide more detail and clearer descriptions of their experiences than health professionals when reporting suspected ADRs indicating a desire to explain their experiences [8, 9].

Besides spontaneous reporting of suspected ADRs, the systematic collection and analyses of postmarketing data concerning the frequency, severity, and types of reported ADRs are of particular importance. Premarketing clinical trials do not have the ability to demonstrate an association between a drug and rare ADRs, which may be observed postmarketing when the drug is used by a larger number of individuals. Furthermore, premarketing safety studies cannot reveal ADRs associated with off-label use of drugs after their availability on the market. Drug and poison information centers (DPIC) can provide timely, quality monitoring and surveillance data on the nature and type of ADRs occurring across the healthcare system and in the community [10]. DPIC pharmacovigilance data are appearing in the medical literature and represent a valuable source of safety surveillance information for public health reporting [11–13].

To broaden the perspective about the overall burden associated with drug-specific ADRs, consideration should be given to their potential in affecting both patients and the health care system, particularly if they result in hospital admissions. Data about the drug-specific ADRs causing hospitalization is limited to some studies. For example, in a prospective multicenter study conducted in the Netherlands [14], drugs associated most often with drug-related hospitalization were those that affect blood coagulation, such as antiplatelets, and oral anticoagulants.

### Aim of the study

The objective of this study was to characterize the epidemiology of reported ADRs and to determine the likelihood of therapeutic group-specific ADRs that result in hospital admissions. To accomplish these objectives, first, we analyzed data from yellow card schemes of reported ADRs to the 13-Aban Drug and Poison Information Center (13-Aban DPIC). The center is the main designated resource for providing medication-use recommendation through telephone management in Tehran. Second, the ADR Hospitalization (ADRH) index was calculated for ADRs with the all implicated therapeutic groups reported to the 13-Aban DPIC.

### Ethical approval

This study used anonymous data that were analyzed on a group basis, and there was no risk to the individuals whose information was in the 13-Aban DPIC database. The study methods and analyses were in accordance with the regular quality-assurance procedures used by the Research Center for Rational Use of Drugs, Tehran University of Medical Sciences.

## Methods

### Study design and setting

We conducted a retrospective analysis of yellow card schemes of the reported ADR incidents to the 13-Aban DPIC from 21 March 2013 to 21 November 2016. The 13-Aban DPIC is a designated recourse for providing recommendations for the medication therapy and onsite treatment of poisoning exposures through telephone consultation in the city of Tehran. The center provides a telephone service that operates from 0800 to 2000 hour daily managing more than 30,000 enquires annually. Beside their primary service aimed to members of the public, the 13-Aban DPIC also provides a separate service to healthcare professionals. The individuals in charge of managing calls are registered pharmacists, physicians, and clinical specialist pharmacists. All enquires to the 13-Aban DPIC are documented on a standardized call report by registered pharmacists or physicians and peer reviewed within hours by clinical specialist pharmacists to ensure accuracy.

### Yellow card scheme

The 13-Aban DPIC follows the definition of an ADR from the World Health Organization (WHO). It was defined as: “a response to a drug which is noxious and unintended, and which occurs at doses normally used in man for the prophylaxis, diagnosis, or therapy of disease, or for the modification of physiological function”[15]. The yellow cards are filled for each reported ADR to drugs, vaccines, herbal or complementary products, whether self-administered or prescribed [16]. The reports are then submitted to the IADRMC at the Ministry of Health.

The data in the yellow card are answers to the 13 questions, listed in Table 1.

**Table 1:** Questions involved in the yellow card scheme.

No.	Question	Descriptions
1	Patient details	Patient initials, age, weight, sex and pregnancy status
2	Type of occurred ADR(s) and any treatment given	-
3	Date ADR(s) started	-
4	Duration ADR(s) lasted	-
5	Patient past ADR(s) history	-
6	Patient medical history	Including patient allergies, congenital diseases, enzyme deficiencies, addiction, and patient current comorbidities
7	Did the ADR disappear after the suspected drug stopped?	Yes /No/ The suspected drug not stopped
8	Did the ADR reappear after the suspected drug readministered?	Yes /No/ The suspected drug were not readministered again
9	The seriousness of occurred ADR	Not a serious ADR and managed onsite/ Serious ADR and involved significant disability or incapacity/ Patient died due to reaction/ Other medically significant outcomes: please give details
10	Did the ADR involve patient hospitalization?	Yes/ No
11	Paraclinical findings associated with the occurred ADR;	-
12	List patient suspected drug(s) that might be associated with occurred ADR	Suspected drug product(s) indicate brand name, if known/ Dosage/ Daily dose/ Route/ Reason(s) for using the product (Indication) / Date started/ Date stopped/ Manufacturer and batch number
13	List all other drug(s) taken by patient at the same time of occurrence of ADR	Drug product(s) indicate brand name, if known/ Dosage/ Daily dose/ Route/ Reason(s) for using the product (Indication) / Date started/ Date stopped/ Manufacturer and batch number.

Abbreviations: ADR, adverse drug reaction.

### Data extraction and analysis

For the purpose of these analyses, data of all ADR-yellow card reports that were submitted to the IADRMC at the Ministry of Health over the study period were included in the study data form. The analytic procedure involved a number of steps. First, the drug related causality was assessed by using Naranjo algorithm [17]. ADRs were classified into definite (score, 9–12 points), probable (score, 5–8 points), possible (score, 1–4 points), or doubtful (score, 0 point). Only definite and probable ADRs were considered for further analysis. Second, several data were extracted from definite and probable ADR-yellow card reports. Data extracted included patient details such as age and sex, the suspected drug associated with ADR, medical outcomes of occurred ADR (minor, moderate or major adverse outcomes), setting of occurred ADR (home-environment and domestic setting or healthcare facilities), and individual who reported ADR to the 13-Aban DPIC (member of the public or healthcare professionals). Third, definite and probable ADR reports associated with the most frequently suspected drug groups were analyzed based on medical outcomes of occurred ADRs or whether patients were hospitalized. Drugs were coded according to the Anatomical Therapeutic and Chemical (ATC) codes [18]. Finally, the ADRH index, a composite measure proposed by the previous study [19], was calculated for suspected drug groups involved with patient hospitalization. ADRH index was calculated by dividing the number of ADR-related hospitalizations for suspected drug groups with the total number of reported ADR cases (n = 748), and then multiplying the number by 100. Hospitalization was defined as need for patient admission. All data management and calculations were performed with SPSS (version 21; SPSS, Inc., Chicago, IL).

### ADR health outcome

Seriousness of medical outcomes associated with ADR were classified based on modified Hartwig and Siegel ADR severity scale [20] and description of adverse events given for each patient in ADR-yellow card into mild, moderate, or major adverse outcomes. Minor adverse outcomes were defined as the patient experiencing minimally bothersome symptoms as a result of the drug use, but that required no change in treatment with the suspected drug. Sometimes the ADR required that the suspected drug be discontinued. No antidote or other treatment was required. Moderate adverse outcomes were defined as the patient experiencing more pronounced and prolonged symptoms as a result of the drug use. The ADR required that the suspected drug be discontinued and an antidote or other treatment be given. Major adverse outcomes were defined as the patient experiencing symptoms that were life-threatening or that resulted in significant disability or incapacity as a result of the drug use. The ADR required intensive medical care.

## Results

13-Aban DPIC received 149,873 calls from 21 March 2013 to 21 November 2016. Of these calls, 781 were identified as an ADR based on the WHO definition for an adverse reaction to drugs and were reported in the yellow card schemes, 748 of which were definite and probable ADR cases.

### ADR report types

Table 2 presents the number of reported ADR cases by calendar year, setting at which reported ADRs occurred, the individual who reported the ADR, and sex and age group of the patient. There were no major fluctuations in the annual distribution of reported ADR cases over the study period. The majority of reported cases of ADRs to 13-Aban DPIC occurred in home-environment and domestic setting (94%, n = 703), but a small number occurred in healthcare facilities (6%, n = 45). Members of the public were predominantly the main source of the ADR reports to the center. They accounted for 87% (n = 651) of all reports, and the remaining 13% (n = 97) of ADRs were reported by health professionals.

**Table 2:** Patient characteristics for reported cases of ADRs.

Characteristics	No. (%) Pts (n = 748)
**Sex**	
** Male**	228 (30.5)
** Female**	520 (69.5)
**Age (yr)**	
** 0–17**	107 (14.3)
** 18–44**	356 (47.6)
** 45–64**	214 (28.6)
** ≥ 65**	71 (9.5)
**Year of exposure**	
**2013**	198 (26.5)
**2014**	262 (35)
**2015**	184 (24.6)
**2016**	104 (13.9)
**Callers identity**	
** Public**	651 (87%)
** Healthcare professionals**	97 (13%)

Abbreviations: ADR, adverse drug reaction.

### Age and sex

Most of the reported ADR cases were female (n = 520, 69.5%). Most of the ADR cases reported to 13-Aban DPIC during the study period were observed in the 18–44 year group (n = 356, 47.6%), and the fewest cases were reported in the elderly (n = 71, 9.5%).

### Drugs identified as ADR suspect

Table 3 and Table 4 shows the major therapeutic groups and specific drug involved in ADR cases reported, respectively. While a total of 298 drugs were identified as suspects, relatively few accounted for most of the ADRs. The 30 antibacterial medications (10%) with the highest ADR totals accounted for 118 of all reported study adverse reactions (15.8%). Among those antibacterial medications, azithromycin (n = 20, 2.7%) and metronidazole (n = 16, 2.1%) had the highest number of ADRs (Table 3). Next being 13 antidepressants (4.3%) with 59 ADRs (7.9%); and finally 10 anticonvulsants (3.3%) with 45 of the reported adverse reactions (6%).

**Table 3:** Most frequent suspected drug classes in ADRs.

Therapeutic group	No. (%)^a^	Minor effect	Moderate effect	Major effect	No. of Pts Admitted to Hospital	ADR Hospitalization Index (%)
**All**	748 (100)	509 (68)	202 (27)	37 (4.9)	55	7.4
**Antibacterials for systemic use**	118 (15.8)	75 (63.6)	31 (26.3)	12 (10.2)	13	1.7
**Antidepressants**	59 (7.9)	43 (72.9)	14 (23.7)	2 (3.4)	3	0.4
**Anticonvulsants**	45 (6)	31 (68.9)	13 (28.9)	1 (2.2)	3	0.5
**Vitamin**, **mineral and herbal supplements**	55 (7.3)	37 (67.3)	16 (29.1)	2 (3.6)	4	0.4
**Anti-neoplastic agents**	31 (4.1)	18 (58.1)	10 (32.3)	3 (9.7)	3	0.5
**Antipsychotics**	29 (3.9)	22 (75.9)	6 (20.7)	1 (3.4)	3	0.5
**Nonstreoidal anti-inflammatory drugs**	25 (3.3)	15 (60)	7 (28)	3 (12)	4	0.4
**Proton pump inhibitors**	21 (2.8)	15 (71.4)	6 (28.6)	0	1	0.13
**HMG-CoA Reductases**	20 (2.7)	10 (50)	10 (50)	0	0	0
**Corticosteroids**	17 (2.3)	12 (70.6)	5 (29.4)	0	0	0
**Anti-diabetic agents**	16 (2.1)	12 (75)	3 (18.8)	1 (6.3)	2	0.3
**Retinoic acid derivatives**	13 (1.7)	8 (61.5)	5 (38.5)	0	0	0
**Calcium channel blockers**	13 (1.7)	11 (84.6)	2 (15.4)	0	0	0
**Anti-histamines for systemic use**	13 (1.7)	8 (61.5)	4 (30.8)	1 (7.7)	1	0.13
**Contraceptives**	11 (1.5)	8 (72.7)	3 (27.3)	0	1	0.13

Abbreviations: ADR, adverse drug reaction; HMG-CoA Reductase: 3-hydroxy-3-methylglutaryl-coenzyme A reductase.

^a^The total number of reported ADRs, n = 748.

**Table 4:** Drug most frequently reported for ADRs.

Drug Name	No. (%)^a^	Drug Class
**All**	298 (100)	
**Azithromycin**	20 (6.7)	Antibacterials for systemic use
**Atorvastatin**	19 (6.4)	HMG-CoA reductase inhibitor
**Metronidazole**	16 (5.4)	Antibacterials for systemic use
**Pantoprazole**	12 (4)	Proton pump inhibitor
**Amlodipine**	12 (4)	Calcium channel blocker
**Isotretinoin**	12 (4)	Retinoic acid derivative
**Co-amoxiclav**	11 (3.7)	Antibacterials for systemic use
**Sertraline**	11 (3.7)	Antidepressant
**Valproate sodium**	11 (3.7)	Anticonvulsant
**Gabapentin**	11 (3.7)	Anticonvulsant
**Cyproterone acetate**	10 (3.3)	Antiandrogen
**Metfomin**	10 (3.3)	Anti-diabetic agent

Abbreviations: ADR, adverse drug reaction; HMG-CoA Reductase: 3-hydroxy-3-methylglutaryl-coenzyme A reductase.

^a^The total number of drug implicated in ADR, n = 298.

### ADR health outcome

Table 3 also presents the medical outcome and the ADRH index for ADR cases with the most frequently implicated therapeutic groups. Most of the ADRs had a medical outcome documented as having a minor outcome (n = 509, 68%), and less than 4.9% (n = 37) were documented as having a major outcome. Overall, 7.4% (n = 55) of ADRs resulted in admission to a hospital. Although antibacterials indicated for systemic use represented the therapeutic group with the highest hospitalization index (1.7%), the reported ADRH index for other drug groups were considered too small for reliable comparisons.

### ADR types

Table 5 shows the most frequent adverse reactions associated with reported ADR cases. Approximately one-fifth of reported ADRs were either associated with central nervous system adverse reactions (n = 171, 23%) or dermatologic adverse reactions (n = 159, 21.4%).

**Table 5:** Report type of adverse drug reactions.

Category	No. (%)^a^	Children and adolescents	Adults^b^	Elderly
**Central nervous system**	171 (23)	21 (12.3)	136 (79.5)	14 (8.2)
**Dermatologic**	159 (21.3)	44 (15.4)	104 (65.4)	11 (6.9)
**Gastrointestinal**	91 (12.2)	14 (15.4)	64 (70.3)	13 (14.3)
**Cardiovascular**	76 (10.2)	3 (3.9)	61 (80.3)	12 (15.8)
**Neuromuscular and skeletal**	49 (6.6)	0	43 (87.8)	6 (12.2)
**Genitourinary**	44 (5.9)	7 (15.9)	34 (77.3)	3 (6.8)
**Ophthalmic**	40 (5.3)	3 (7.5)	32 (80)	5 (12.5)
**Hypersensitivity**	36 (4.8)	7 (19.4)	29 (80.6)	0
**Endocrine and metabolic**	23 (3.1)	1 (4.3)	19 (82.6)	3 (13)
**Respiratory**	21 (2.8)	4 (19)	16 (76.2)	1 (4.8)

Abbreviations: ADR, adverse drug reaction; HMG-CoA Reductase: 3-hydroxy-3-methylglutaryl-coenzyme A reductase.

^a^The total number of reported ADRs, n = 748.

^b^All adults aged 18–64 years.

## Discussion

The present study provides insight into the nature and type of ADRs that occur in domestic settings in Tehran, Iran. This study analyzed data from 748 cases of yellow cards of suspected ADRs reported to a major regional DPIC in the city of Tehran. Numerous studies have tried to assess the patterns and nature of ADRs occurring in ambulatory and out-patient settings [21, 22], however, this study might be the first to use data from yellow card schemes of suspected ADRs reported directly by the members of the public which makes the data more representative of the nature of ADRs that occur in the home-environment on everyday basis.

The current study found that ADRs accounted for 5 cases per 1000 enquiries in the study period of almost 4 years. This proportion is lower than that reported by the American Poison Control Center with approximately 17 cases per 1000 of enquiries relating to ADRs occurring in one year, 2015 [23]. We also found that the majority (87%) of ADRs were reported directly by the members of public, thus, compared to the incidents of ADRs from the American Poison Control Center, the low number of ADR reports from health professionals (13%) to our DPIC might be the reason behind the lower incident of ADR reports. Individual hospitals often collect data on ADR incidents for their own institution and directly report them to ADR monitoring center and there is no obligation on the clinician to contact the drug information centers for advice or to report ADR incidents. Although these DPICs are ideally suited to provide clinical advice or guidance to healthcare professionals following occurrence of an adverse reaction to a drug therapy [13], this role is not yet widely recognized.

In this study we received ADR cases reported from different age groups. We found that more than 70% of reported ADRs involved adults, 9% involved elderly (65-years old), and 14% involved children and adolescents. The observed bell-shaped pattern of ADRs in different age groups in our study are likely related to the expansive population pyramid (young and growing) of Iran [24], with more ADRs occurring in adults. Another explanation of the current results could be that compared to the other age groups, adults may have had more tendency to contact our DPIC and therefore were associated with more ADR case reports. Unlike other studies [25, 26], our analysis did not identify elderly as being at high risk of developing ADRs. Elderly may have concurrent comorbid conditions that require complex medical regimens [25], and the co-administration of multiple medications can lead to drug-drug interactions that increases their possibility of developing ADRs [27]. Furthermore, few studies to date have investigated risk factors for ADRs in children. In the systematic review by Smyth et al, female gender, increasing number of drugs, off-label use and oncological treatment were identifiable risk factors. The same review also highlighted that anti-infective drugs were among the most therapeutic groups associated with ADRs in children out-patient setting [28]. Similarly, we also found that anti-infective medications were the most frequent therapeutic group associated with the occurrence of ADRs in children and within all age groups. We strongly recommend further effort in addressing prescription practices in different settings to prevent ADRs.

A novel and noteworthy finding in our study relates to the use of the DPIC data in evaluating the burden associated with ADRs. We found that 7% of reported ADRs led to hospital admission. Likewise, in a retrospective analysis of ADR cases reported to the New Jersey Poison Information and Education System (NJPIES) [19], 7% of reported ADR leads to patient hospital admission, and antidepressants were the most implicated drugs to cause ADR-related hospitalization. We also attempted to describe and compare the likelihood of ADR-related hospitalizations for different therapeutic groups based on the data delivered from ADR yellow card reports. The ADRH index which was calculated for all therapeutic groups suggests that the burden associated with ADRs differs among therapeutic classes. While antibacterials and antidepressants were the most commonly implicated groups in ADRs, antibacterials had the highest probability for an ADR-related hospital admission and had the highest ADRH index. We suggest that although many of the suspected drugs have proven clinical benefits, efforts should be made to improve their overall benefit–burden balance.

There are several limitations of the data of this study that have to be acknowledged. First, the records of our center are largely based on self-reported medication use and adverse outcome information, which in most cases can only be confirmed if the patient is followed up to a healthcare facility. Second, the analyses did not evaluate the effect of patient preexisting medical and comorbid conditions that may have played a role in the observed associations. Third, we believe that despite limitations this study provides essential information about the likelihood of therapeutic group-specific ADRs to result in hospitalization. Nonetheless, despite these limitations, our data provide insights into the nature of ADRs reported to DPIC which built the foundation for future research. This information can be used by healthcare providers and pharmaceutical companies to develop targeted strategies for reducing the number of ADRs to drugs with a high ADRH index.

## Conclusions

In our study we have shown the patterns and nature of ADRs occurring in home-environment setting; we found the majority of ADR cases were reported directly by the member of public. We found that 7% of reported ADRs led to hospital admission. Among the therapeutic groups that were most frequently implicated in ADRs, antibacterials for systemic use showed the highest probability for an ADR-related hospitalization.

## Supporting information

S1 File Study raw data. (SAV)
